# Capture rates of *Eptesicus fuscus* increase following white‐nose syndrome across the eastern US

**DOI:** 10.1002/ece3.11523

**Published:** 2024-06-25

**Authors:** Molly C. Simonis, Lynn K. Hartzler, Gregory G. Turner, Michael R. Scafini, Joseph S. Johnson, Megan A. Rúa

**Affiliations:** ^1^ Environmental Sciences PhD Program Wright State University Dayton Ohio USA; ^2^ School of Biological Sciences University of Oklahoma Norman Oklahoma USA; ^3^ Department of Biological Sciences Wright State University Dayton Ohio USA; ^4^ Pennsylvania Game Commission Bureau of Wildlife Management Harrisburg Pennsylvania USA; ^5^ School of Information Technology University of Cincinnati Cincinnati Ohio USA

**Keywords:** big brown bat, *Eptesicus fuscus*, introduced pathogen, *Pseudogymnoascus destructans*, white‐nose syndrome, wildlife populations

## Abstract

Emerging infectious diseases threaten wildlife globally. While the effects of infectious diseases on hosts with severe infections and high mortality rates often receive considerable attention, effects on hosts that persist despite infection are less frequently studied. To understand how persisting host populations change in the face of disease, we quantified changes to the capture rates of *Eptesicus fuscus* (big brown bats), a persisting species susceptible to infection by the invasive fungal pathogen *Pseudogymnoascus destructans* (*Pd*; causative agent for white‐nose syndrome), across the eastern US using a 30‐year dataset. Capture rates of male and female *E. fuscus* increased from preinvasion to pathogen establishment years, with greater increases to the capture rates of females than males. Among females, capture rates of pregnant and post‐lactating females increased by pathogen establishment. We outline potential mechanisms for these broad demographic changes in *E. fuscus* capture rates (i.e., increases to foraging from energy deficits created by *Pd* infection, increases to relative abundance, or changes to reproductive cycles), and suggest future research for identifying mechanisms for increasing capture rates across the eastern US. These data highlight the importance of understanding how populations of persisting host species change following pathogen invasion across a broad spatial scale. Understanding changes to population composition following pathogen invasion can identify broad ecological patterns across space and time, and open new avenues for research to identify drivers of those patterns.

## Graphical abstract

To understand how persisting host populations change in the face of disease, we quantified changes to the capture rates of *Eptesicus fuscus* (big brown bats), a species that is annualy exposed and infected by the invasive fungal pathogen *Pseudogymnoascus destructans* (*Pd*); causative agent for white‐nose syndrome), using a 30‐year dataset across the eastern US. Here, the overall capture rates of adult *E. fuscus* increased from preinvasion to *Pd* establishment years across the eastern US. In conclusion, our work highlights the need to better understand how host populations change across broad spatial scales with continued pathogen exposure.

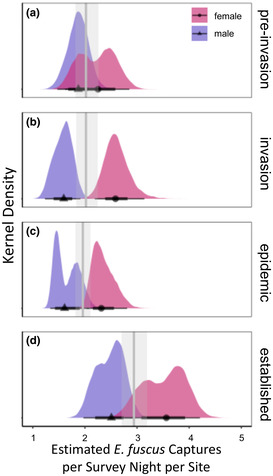

## 1 INTRODUCTION

Emerging infectious diseases threaten wildlife populations (Daszak et al., 2000; Young et al., 2017). Changes to wildlife populations following disease emergence can be a result of spatiotemporal pathogen spread and heterogeneity of host responses to infection (e.g. physiological or behavioral) across varying landscapes (Becker et al., 2020; Hawley & Altizer, 2011; Kailing et al., 2023; Lopes et al., 2021; Simonis, Hartzler, Turner, et al., 2023). These responses can also differentiate across demographics within a population due to differences in prevalence, infection intensity, and/or survival between male and female hosts (Kailing et al., 2023; Retschnig et al., 2014; Russell et al., 2019). Quantifying these changes within wildlife populations at smaller scales is important for local conservation and management actions. However, understanding how wildlife populations change across broad spatial scales following disease emergence can help identify regional research needs for understanding species survival and/or persistence.

North American bat populations are currently threatened by *Pseudogymnoascus destructans* (*Pd*), an invasive fungal pathogen from Eurasia that causes white‐nose syndrome (Blehert et al., 2009; Drees et al., 2017; Hoyt et al., 2016; Lorch et al., 2011). *Pd* was first detected in New York, USA, in 2006 and has since spread across North America (Blehert et al., 2009; White Nose Syndrome Response Team, 2022). *Pd* infects skin tissues of hibernating North American bats and causes increased evaporative water loss, hypotonic dehydration, increased arousals from torpor, and increased torpid metabolic rates, depleting their fat stores faster than without infection during hibernation (Cryan et al., 2013; McGuire et al., 2017; Moore et al., 2018; Reeder et al., 2012). These devastating physiological responses have resulted in mortality rates in the eastern US of more than 90% in species that are highly susceptible to severe infections such as *Myotis lucifugus* (little brown bat), *Myotis septentrionalis* (Northern long‐eared bat) and *Perimyotis subflavus* (tri‐colored bat; Cheng et al., 2021, Frick et al., 2015). Furthermore, increased energy expenditures and declines to host populations that obtain severe infections are also reflected in months outside of winter (Dzal et al., 2011; Meierhofer et al., 2018; Moosman et al., 2013). While the impacts of *Pd* infection devastate many hibernating North American bat populations, how this pathogen changes demographic compositions of hosts that persist despite infection are unclear.

North American populations of *Eptesicus fuscus* (big brown bat) have estimated declines of 35%–41% since the introduction of *Pd* and thus, persist in larger numbers compared to host species that obtain severe infections (Cheng et al., 2021; Turner et al., 2011). With some degree of resistance to *Pd* infections (Frank et al., 2014; Moore et al., 2018), *E. fuscus* maintains lower *Pd* prevalence, infection intensities and disease severity compared to species susceptible to severe infections during hibernation (Langwig, Frick, et al., 2015; Moore et al., 2018). However, unlike host species that have greater populations losses, summer capture rates of *E. fuscus* have variable changes following *Pd* introduction (Francl et al., 2012; Moosman et al., 2013; O'Keefe et al., 2019; Pettit & O'Keefe, 2017). Due to mismatched trends between winter population losses and variable summer capture rates, understanding how *E. fuscus* population compositions differ when bats are active in spring through fall months following *Pd* introduction is important for estimating the future success of persisting populations—especially since winter hibernacula surveys cannot account for changes in female reproductive demographics.

At spatially smaller scales, some sites in the eastern and midwestern US have increased capture rates of *E. fuscus* following *Pd* introduction (Francl et al., 2012; O'Keefe et al., 2019; Pettit & O'Keefe, 2017). Furthermore, post‐lactating female *E. fuscus* captures rates increased in the Smokey Mountains of the US (O'Keefe et al., 2019), nonreproductive female bat capture rates increased in Indiana, US (Pettit & O'Keefe, 2017), and reproductive female capture rates did not change in West Virginia, US following *Pd* introduction (Francl et al., 2012). To better understand how persisting *E. fuscus* populations may be responding to the physiological consequences of long‐term *Pd* exposure, we need to examine broad spatial patterns for changes in their capture rates over time, and outside of hibernating months with *Pd* infections.

To determine how *E. fuscus* populations differentially change across a broad spatial scale over time since pathogen introduction, we quantified changes to capture rates using a 30‐year dataset of individual *E. fuscus* captures in spring through fall months across the eastern US (Simonis et al., 2022; Simonis, Hartzler, Turner, et al., 2023). We hypothesized that the capture rates of adult male and female *E. fuscus* would change across the eastern US with increasing *Pd* exposure time and latitude, and that those changes would depend on bat demographics (i.e., sex or female reproductive status). Here, we highlight the importance of understanding how pathogen introductions may change persisting host populations and their compositions over pathogen invasion time and a broad spatial scale, and target future research questions for identifying mechanisms of *E. fuscus* persistence.

## 2 METHODS

We used a large, publicly available dataset of adult *E. fuscus* capture records spanning 30 years across 11 US states (GA, IL, IN, KY, MS, NY, NC, OH, PA, TN, and VA), which were collected from government wildlife agencies and bat researchers in the eastern US (Simonis et al., 2022; Simonis, Hartzler, Campbell, et al., 2023; Simonis, Hartzler, Turner, et al., 2023). We used adult *E. fuscus* records from this dataset which consisted of 24,129 individual bats (females *n* = 14,162; males *n* = 9967) captured across 3567 sites outside of hibernating months in April through October from 1990 to 2020 (Figure S1; Simonis et al., 2022; Simonis, Hartzler, Campbell, et al., 2023; Simonis, Hartzler, Turner, et al., 2023). Variables within the data used for this paper were: date of capture, capture site name, county centroid latitude of capture (ranging from 30.5° N to 44.8° N), sex (male/female) and reproductive status (females only: nonreproductive/pregnant/lactating/post‐lactating). We also used variables for standardized *Pd* invasion time across each state and year of capture which were classified by *Pd* exposure time‐steps (Cheng et al., 2021; Langwig, Voyles, et al., 2015; Simonis, Hartzler, Campbell, et al., 2023; Simonis, Hartzler, Turner, et al., 2023). Preinvasion years were those before suspected or confirmed *Pd* introduction in each state (<0 years), invasion years occurred 0–1 years within documented *Pd* introduction in each state, epidemic years were designated as those 2–4 years following *Pd* introduction in each state, and established years occurred 5+ years after *Pd* introduction in each state.

The number of captures per survey night per site were used to standardize capture per unit effort across all 3567 capture sites. We calculated capture rates (captures per survey night per site) by first quantifying the number of consecutive dates a site was visited using the package *dplyr* in R version 4.3.1 (R Core Team, 2023; Wickham et al., 2021). We then summarized individual capture records for adult *E. fuscus* within the dataset by the number of individuals captured per consecutive survey dates per site within each state and county of capture, sex, and reproductive status using the package *dplyr* in R version 4.3.1 (R Core Team, 2023, Wickham et al., 2021). Finally, we divided the conditional number of individuals captured per survey per site by the total number of consecutive dates within each survey to obtain captures per survey night per site.

To determine how time since *Pd* invasion changed the capture rates of adult *E. fuscus* by sex and reproductive status (female only) across space, we used a full adult dataset to compare female and male capture rates and a female‐only dataset to compare changes in capture rates by reproductive demographics (Simonis, Hartzler, Campbell, et al., 2023; see also appendix S1: fig. S1 in Simonis, Hartzler, Turner, et al., 2023 for total capture counts per number of sites by *Pd* time‐steps and sex). Our analyses only considered reproductive status for female *E. fuscus* because females move through reproductive life stages in spring through fall months, which coincides with the collection of the capture records used here, while male reproduction does not occur until fall or winter, which is outside the collection period of this data.

### 2.1 Statistical analyses

We completed all statistical analyses in R version 4.3.1 and data visualizations were made using the packages *ggplot2*, *tidybayes*, and *patchwork* (Kay, 2020; Pedersen, 2020; R Core Team, 2023; Wickham, 2016). To determine how the capture rates of *E. fuscus* changed across the eastern US over *Pd* invasion time‐steps, we used a two‐step modeling approach (Figure S2). We created separate Bayesian generalized linear mixed models for all adult *E. fuscus* (males and females combined) or only female *E. fuscus* at both model creation steps. All models were created using STAN computational framework R interface (*rstan* package) with function *brm* in the *brms* package (Bürkner, 2017; Stan Development Team, 2020). Models were run remotely using the University of Oklahoma's (OU) supercomputer via the OU Supercomputing Center for Education and Research.

Initial models were created to describe how the capture rates of *E. fuscus* sex or female reproductive statuses changed with latitude over disease time‐steps (Figure S2). These Bayesian generalized linear mixed models were created with Gamma distributions for bat capture rates (capture counts per survey night per site). While capture rate data had a negative binomial distribution, we used a Gamma family to account for noninteger capture rates. To determine how adult *E. fuscus* capture rates changed by sex with latitude and *Pd* exposure time‐steps, we created an initial model for adult bat capture rates (males and females combined) as a function of a conditional effects interaction for sex, latitude of capture county centroid and *Pd* exposure time‐steps (Figure S2a). To determine how female *E. fuscus* capture rates changed by reproductive status due to latitude and *Pd* exposure time‐steps, we created an initial model for the capture rates of female bats as a function of a conditional effects interaction for reproductive status, latitude of capture county centroid, and *Pd* exposure time‐steps (Figure S2b). To additionally account for varying effort in site visits, we also included capture site as a group level effect in initial models (Figure S2).

We used weakly informed prior distributions for adult and female‐only models separately using a normal distribution with a mean of 0 and a standard deviation of 10. We also used a Gamma shape and scale of 0.01, representative of weakly‐informed negative binomial shape. Both initial adult and female‐only models were each run on four Markov chains at 10,000 iterations with a burn‐in period of 5000 iterations. Both adult and female‐only models converged with Ȓ values at 1.00 and resulted in 160,000 posterior samples for adult models and 320,000 posterior samples for female‐only models. The larger posterior samples for the female‐only model compared to the adult model reflects the number of levels within the conditional effects interaction. Specifically, in the adult model (Figure S2a), sex has two levels (male/female) while in the female‐only model (Figure S2b), reproductive status has four levels (nonreproductive/pregnant/lactating/post‐lactating). Finally, in order to determine how *Pd* exposure time‐steps altered *E. fuscus* capture rates across space, we calculated the slopes for capture rates across latitude for each time‐step by sex for adult models (Figure S2a) or reproductive status for female models (Figure S2b) using the *emtrends* function in the *emmeans* package (Lenth, 2021).

**FIGURE 1:**
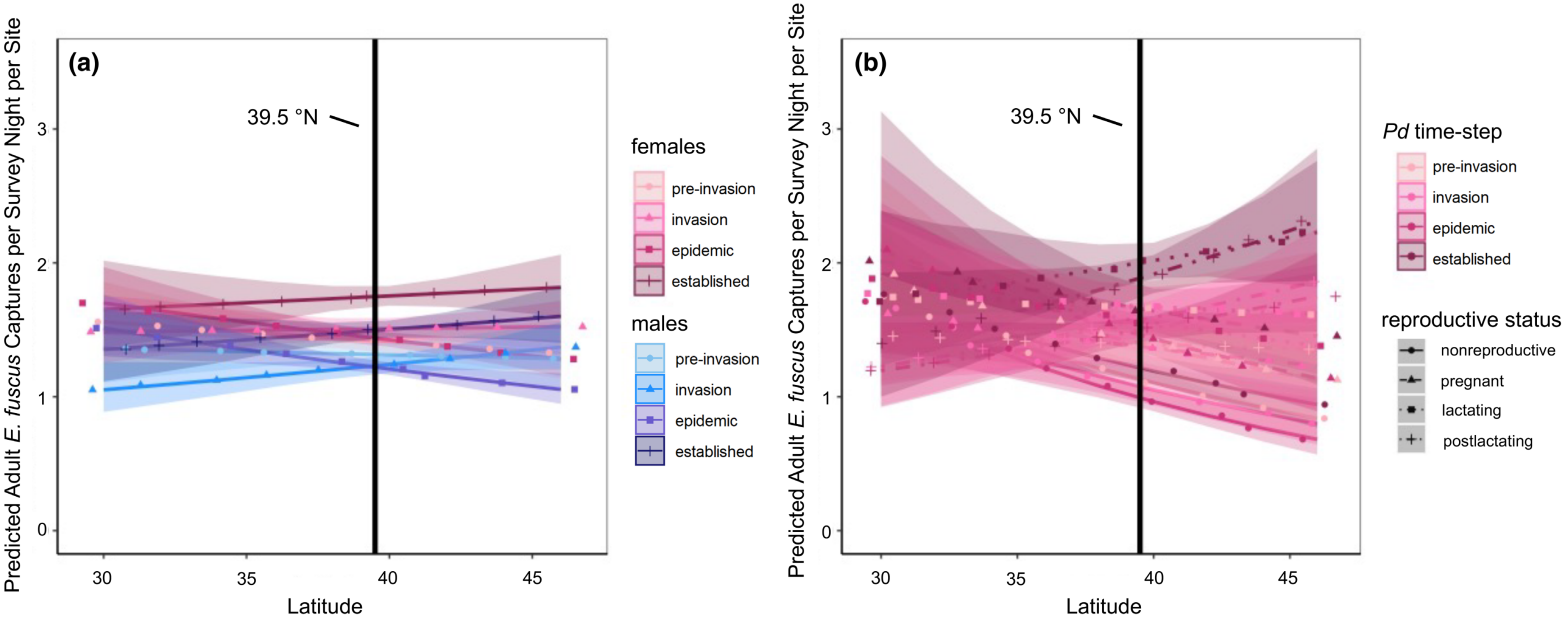
Demographic changes to the capture rates of *E. fuscus* occurred over *Pseudogymnoascus destructans* (*Pd*) exposure time‐steps and latitude for adults [*R* ^2^ = 0.16 (0.14, 0.17); a] and females‐only [*R* ^2^ = 0.17 (0.15, 0.19); b]. The interaction of *Pd* time‐steps, latitude and sex (a) or female reproductive status (b) occurred at 39.5° N for both models (vertical black line). Capture r south of spatial thresholds varied compared to values north of spatial thresholds. Points are predicted posterior mean estimates for whole number latitudes from 30 to 45° N. Lines represent predicted values for posterior mean estimates with shaded 95% credible intervals. Color reflects *Pd* exposure time‐steps for males (blue hues) or females (pink hues), and line type reflects reproductive status (females only, a). *Pd* time‐steps were categorical for the number of years since suspected or confirmed *Pd* introduction within each state of capture and included preinvasion (<0 years with *Pd*), invasion (0–1 years with *Pd*), epidemic (2–4 years with *Pd*) and established years (5+ years with *Pd*).

Initial models for both adults (Figure S2a: Model 1) and females‐only (Figure S2b: Model 1) indicated a spatial threshold at 39.5° N where the interaction of capture county centroid latitude, *Pd* exposure time‐steps and sex (adult model, Figure S2a) or reproductive status (female‐only model, Figure S2b) occurred (Figure 1). Therefore, we created a second model for adults or females‐only to better understand how capture rates changed above and below the identified spatial threshold over *Pd* exposure time‐steps. To do so, we created a variable for geographic region within the full adult and female‐only datasets for ‘north’ and ‘south’ of 39.5° N. The second Bayesian generalized linear mixed models were then created in the same way as initial models, but we replaced county centroid latitude with the newly created variable for regions “north” and “south” of the identified spatial threshold (Figure S2: Model 2). Like in initial models, capture site was used as a group level effect for both adult and female‐only models (Figure S2: Model 2).

Both models were set on the same prior distribution as first set of models: a normal distribution with a mean of 0, a standard deviation of 10, and a Gamma shape and scale of 0.01. Like initial models, the second adult and female‐only models were each run on four Markov chains at 10,000 iterations with a burn‐in period of 5000 iterations. Adult and female‐only models converged with Ȓ values at 1.00, and resulted in 320,000 posterior samples for adults and 640,000 posterior samples for females. Similar to the previous models, the larger posterior samples for the second model compared to the first model reflects the replacement of the continuous variable for county centroid latitude for the categorical variable with levels for “north” and “south” of the spatial thresholds. Thus, posterior samples doubled to include posterior values for both levels.

To understand how the capture rates of adult *E. fuscus* changed by sex (Figure S2a) and how the capture rates of female *E. fuscus* changed by reproductive status (Figure S2b) over invasion time‐steps and region, we extracted posterior means and credible intervals using *emmeans* function in the package *emmeans* (Lenth, 2021), and made comparisons for *Pd* exposure time‐steps and region by sex (adult model) or reproductive status (female model).

## 3 RESULTS

### 3.1 Changes in adult *E. fuscus* capture rates by sex

Changes in the capture rates of adult *E. fuscus* varied across a spatial threshold at 39.5° N over *Pd* exposure time‐steps and depended on sex [mean (95% credible intervals): *R* ^2^ = 0.16 (0.14, 0.17); Figure 1a and Figure S3]. The capture rates of adult female bats were similar across latitude in preinvasion years [mean slope (95% credible intervals): −0.01 bats per survey night per site (−0.03, 0.01)] and invasion years [−0.004 bats per survey night per site (−0.03, 0.02)], decreased across latitudes in epidemic years [−0.02 bats per survey night per site (−0.03, −0.003)], and stabilized again in establishment years [0.01 bats per survey night per site (−0.01, 0.03); Table S1, Figure 1]. The capture rates of adult male *E. fuscus* changed similarly across *Pd* invasion time‐steps, with similar capture rates with latitude in preinvasion [−0.004 bats per survey night per site (−0.03, 0.02)] and invasion years [0.02 bats per survey night per site (−0.001, 0.04)], decreasing capture rates across latitudes in epidemic years [−0.02 bats per survey night per site (−0.04, −0.01)], and stabilization with latitude by *Pd* establishment [0.01 bats per survey night per site (−0.01, 0.03); Table S1, Figure 1].

**FIGURE 2:**
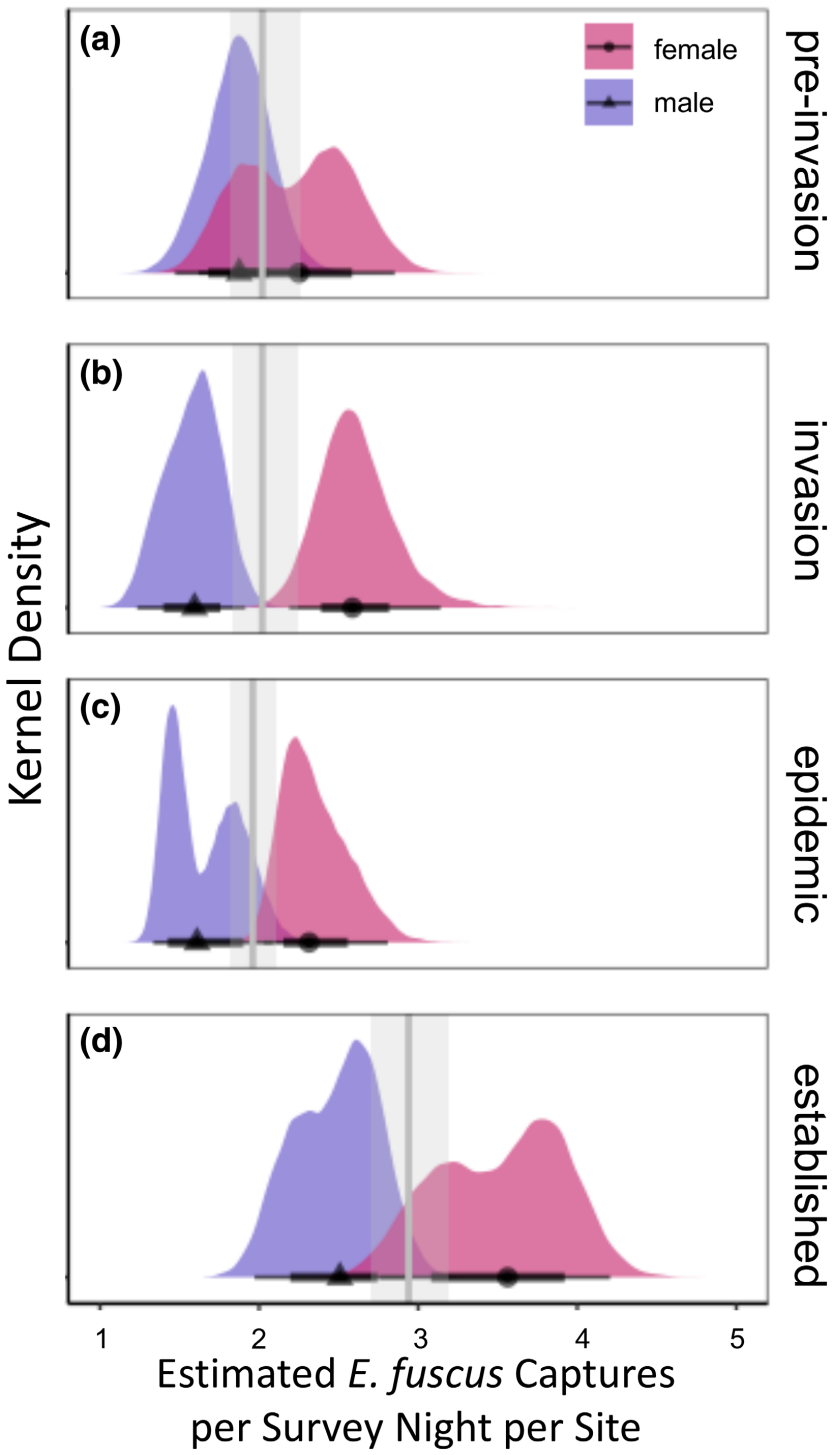
The capture rates of adult *E. fuscus* increased throughout *Pd* time‐steps of preinvasion (a), invasion (b), epidemic (c) and establishment years (d) [*R* ^2^ = 0.16 (0.14, 0.17)]. Distributions of estimated male bat capture rates are represented in purple and female bat capture rates in pink. Black points represent the posterior mean for the capture rates of male (triangles) or female (circles) *E. fuscus*, thick black bars represent 66% credible intervals, and thin black bars are 95% credible intervals. Vertical gray lines are the posterior mean for each *Pd* time‐step. Gray transparent rectangles represent 95% credible intervals around the posterior mean for each Pd time‐step. Significant differences are where there is no overlap in 95% credible intervals.

The capture rates of adult *E. fuscus* varied north to south of 39.5° N by sex over *Pd* exposure time‐steps [*R* ^2^ = 0.16 (0.14, 0.17); Figure 2 and Figure S4]. Neither males nor females differed by capture rates between northern and southern regions (Table S2), but there were significant differences in bat capture rates over invasion time‐steps. Overall, the capture rates of bats increased from preinvasion to established years [mean estimate (95% credible intervals): preinvasion: 2.02 bats per survey night per site (1.81, 2.26), established: 2.94 bats per survey night per site (2.70, 3.19)], and this pattern held for both sexes [preinvasion female: 2.20 bats per survey night per site (1.93, 2.51), established female: 3.50 bats per survey night per site (3.15, 3.89); preinvasion male: 1.86 bats per survey night per site (1.62, 2.13), established male: 2.47 bats per survey night per site (2.22, 2.74); Figure 2a,d; Table S2]. The capture rates of female *E. fuscus* were increasing and males decreasing during invasion years [invasion: 2.02 bats per survey night per site (1.83, 2.24), invasion female: 8.11 (7.06, 9.32), invasion male: 1.57 (1.39, 1.78); Figure 2b], but the capture rates of male or female *E. fuscus* was never different than the average capture rates for each individual time‐step at any other time (Figure 2a,c,d; Table S2).

While the capture rates of all adult *E. fuscus* increased from preinvasion to establishment years, the capture rates of female *E. fuscus* increased more than the capture rates of male *E. fuscus* by *Pd* exposure time‐steps (Figure 2). In preinvasion years, the capture rates of male and female *E. fuscus* were not significantly different from one another [preinvasion female: 2.20 bats per survey night per site (1.93, 2.51), preinvasion male: 1.86 bats per survey night per site (1.62, 2.13); Figure 2a, Table S2]. However, by *Pd* establishment, the capture rates of female *E. fuscus* were almost 1.5 times greater than the capture rates of male *E. fuscus* [established female: 3.50 bats per survey night per site (3.15, 3.89), established male: 2.47 bats per survey night per site (2.22, 2.74); Figure 2d, Table S2].

### 3.2 Changes in female *E. fuscus* capture rates by the reproductive status

Changes in the capture rates of female *E. fuscus* varied across a spatial threshold at 39.5° N over *Pd* exposure time‐steps and depended on reproductive status [*R* ^2^ = 0.17 (0.15, 0.19); Figure 1b and Figure S5]. Capture rates of nonreproductive female bats decreased across latitudes in preinvasion [mean slope (95% credible intervals): −0.05 bats per survey night per site (−0.07, −0.02)], invasion [−0.05 bats per survey night per site (−0.07, −0.02)], epidemic [−0.06 bats per survey night per site (−0.08, −0.03)] and establishment years [−0.04 bats per survey night per site (−0.07, −0.01); Table S1, Figure 1b]. The capture rates of pregnant *E. fuscus* were did not change across latitudes in preinvasion years [−0.03 bats per survey night per site (−0.07, 0.00)] and invasion years [−0.02 bats per survey night per site (−0.06, 0.02)], but decreased with latitude in epidemic [−0.04 (−0.07, −0.01)] and establishment years [−0.02 bats per survey night per site (−0.06, −0.02); Table S1, Figure 1b]. Lactating bat capture rates remained similar across latitudes in preinvasion [0.00 bats per survey night per site (−0.04, 0.03)], invasion [−0.01 bats per survey night per site (−0.04, 0.02)] epidemic [−0.02 (−0.04, 0.01)], and *Pd* establishment years [0.02 bats per survey night per site (−0.02, 0.05); Table S1, Figure 1b]. Finally, the capture rates of post‐lactating bats were similar across latitudes in preinvasion years [0.00 bats per survey night per site (−0.03, 0.03)], but increased with latitude during invasion [0.03 bats per survey night per site (0.00, 0.05)], epidemic [0.02 bats per survey night per site (0.00, 0.05)] and establishment years [0.03 bats per survey night per site (0.00, 0.06); Table S1, Figure 1b].

**FIGURE 3:**
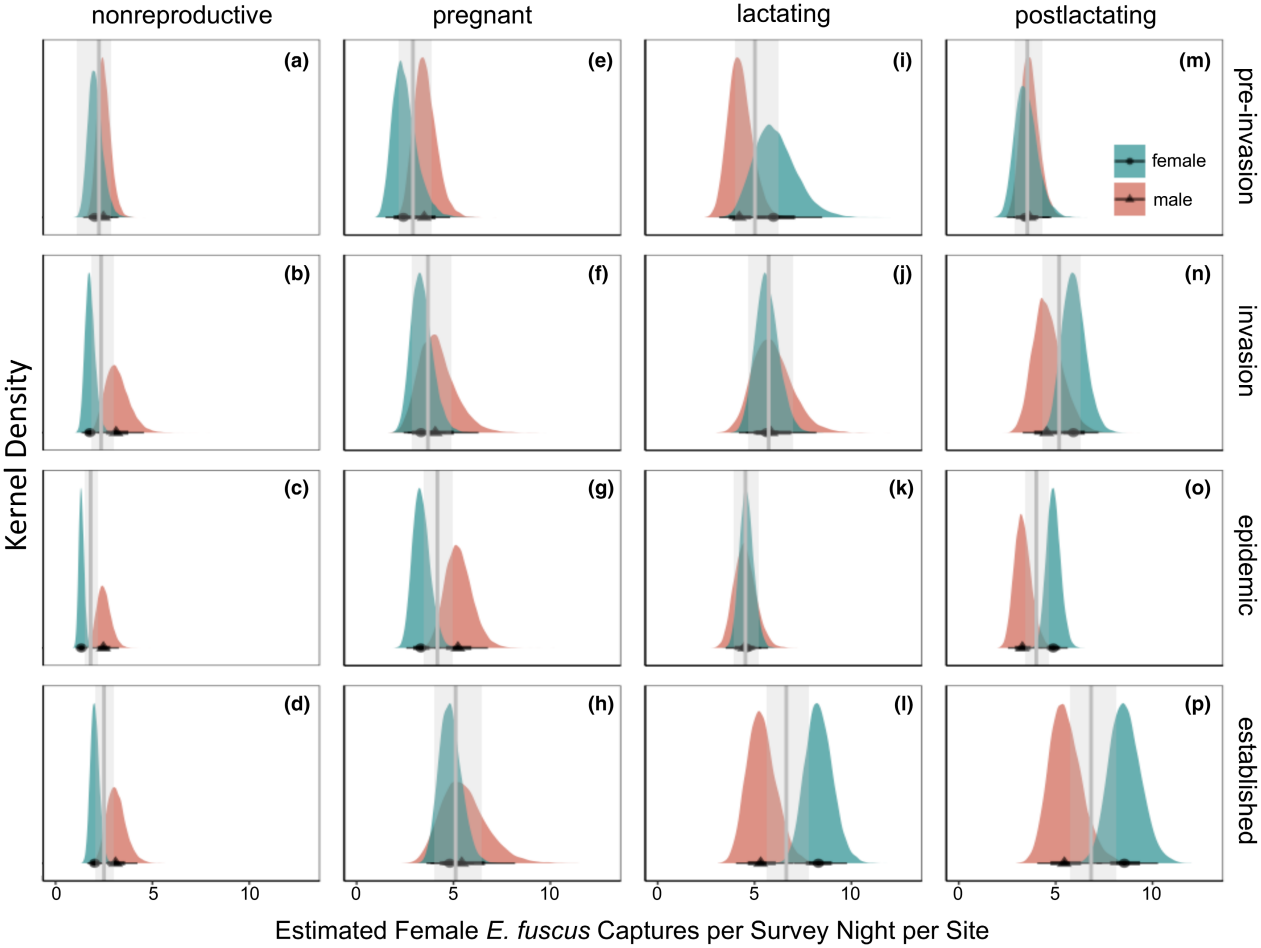
Capture rates varied for nonreproductive (a–d), pregnant (e–h), lactating (i–l), and post‐lactating *E. fuscus* females (m–p) with *Pd* exposure time‐steps and region [north/south; *R* ^2^ = 0.17 (0.15, 0.19)]. Northern female bat capture rates are represented in blue and southern female bat capture rates in red. Black points represent the posterior mean for northern (circles) or southern (triangles) *E. fuscus* capture rates, thick black bars represent 66% credible intervals, and thin black bars are 95% credible intervals. Vertical gray lines are the posterior mean for each *Pd* time‐step within a given reproductive status. Gray transparent rectangles represent credible intervals around the posterior mean for each *Pd* time‐step within a given reproductive status. Significant differences are where there is no overlap in 95% credible intervals.

The capture rates of female *E. fuscus* varied by region, *Pd* exposure time‐steps and reproductive status [*R* ^2^ = 0.18 bats per survey night per site (0.17, 0.20); Figure 3 and Figure S6]. Distinctive shifts between northern and southern nonreproductive female bat capture rates were only prevalent in capture rates of nonreproductive bats (Figure 3a–d, Table S2). Specifically, the capture rates of nonreproductive female *E. fuscus* were not different in the north or south in preinvasion, invasion and establishment years, but northern capture rates were less than southern capture rates during epidemic years [mean estimate (95% credible intervals): nonreproductive epidemic north: 1.33 bats per survey night per site (1.09, 1.63), nonreproductive epidemic south: 2.47 bats per survey night per site (1.87, 3.27); Figure 3c, Table S2].

Pregnant bat capture rates almost doubled from preinvasion to established years [pregnant preinvasion: 2.91 bats per survey night per site (2.17, 3.90), pregnant established: 5.11 bats per survey night per site (3.99, 6.44); Figure 3e,h, Table S2], but did not have differences between northern and southern regions across *Pd* time‐steps (Figure 3e–h). Lactating bat capture rates did not change over *Pd* invasion time steps, nor did they differentiate across the latitudinal threshold during any time. Finally, capture rates of post‐lactating bats almost doubled from preinvasion to establishment years [post‐lactating preinvasion: 3.54 bats per survey night per site (2.88, 4.34); post‐lactating established years: 6.83 bats per survey night per site (5.72, 8.12); Figure 3m,p, Table S2].

## 4 DISCUSSION

While emerging infectious diseases can drive wildlife species to, or near, extinction, understanding the impacts of disease on persisting hosts can open insights to survivability and/or indirect consequences of pathogen exposure. Here, we broadly identified temporal and some spatial relationships between persisting bat capture rates across pathogen invasion time within the white‐nose syndrome/*E. fuscus* system. The overall capture rates of persisting adult *E. fuscus* increased from preinvasion to *Pd* establishment years across the eastern US. Increases in the capture rates of female bats were greater than increases in the capture rates of male bats. There were also general increases to pregnant and post‐lactating female *E. fuscus* capture rates over *Pd* invasion time. This work informs how the intraspecific demographic composition of persisting hosts can change across a broad spatial scale following pathogen invasion. Here, we outline possible mechanisms that could contribute to these patterns, and future research avenues that aim to better understand how *E. fuscus* populations persist despite long‐term exposure to *Pd*.

### 4.1 Energy deficits as a possible mechanism for increased capture rates

Cumulative energy deficits caused by annual *Pd* infections year after year could contribute to the differential increases between male and female capture rates since disease emergence. While overall adult *E. fuscus* capture rates increased from preinvasion to establishment years, male: female capture ratios decreased over *Pd* exposure time‐steps because increases to female capture rates outpaced males. In preinvasion years, the capture rates of male and female bats per site per year was indistinguishable. However, by *Pd* establishment, increases to male capture rates averaged a bat (1.03) less than increases to female capture rates [established female: 3.50 bats per survey night per site (3.15, 3.89), established male: 2.47 bats per survey night per site (2.22, 2.74)], with the difference between male and female bat capture rates across the eastern US being 4566 bats per survey night when extrapolated across 3567 unique sites. During spring and summer months, female *E. fuscus* reproduce (Kurta & Baker, 1990), enduring additional energy costs following winter *Pd* infections. Therefore, energy deficits for female *E. fuscus* in spring through fall months are greater than males because they are recovering from *Pd* infections and reproducing at the same time. Energy deficits from solely healing *Pd* infections in spring are known to occur in *M. lucifugus,* with increased energy expenditures after emerging from hibernation (Meierhofer et al., 2018). Greater energy deficits in females during reproduction could drive greater increases to foraging and thus, capture rates, compared to male *E. fuscus,* as well as other temperate bat species with winter *Pd* infections. To determine the extent of energy deficits *E. fuscus* acquires annually, whole organism energy expenditures need to be measured for male and female bats at varying times, reproductive stages, and ambient temperatures in indi infected vs noninfected individuals.

### 4.2 Ecological pressures as a possible mechanism for increased capture rates

Populations of bat host populations devastated by *Pd* infections and white‐nose syndrome are well documented, with losses represented all year round (Cheng et al., 2021; Dzal et al., 2011; Moosman et al., 2013). Alternatively, winter declines in *E. fuscus* populations due to *Pd* infections are mismatched with local scale summer capture rates, which can be used as a relative abundance metric (Conroy & Nichols, 1996; Kunz et al., 2009). Because *E. fuscus* are known to frequently move between hibernacula in winter and/or hibernate in man‐made structures that are not regularly surveyed (Boyles et al., 2007; Mills et al., 2019; Rysgaard, 1942; Swanson & Evans, 1936; Whitaker & Gummer, 1992, 2000), estimates for how their populations change due to *Pd* may not be best represented through winter hibernacula surveys. Therefore, if spring through fall capture rates are proportional to abundance following disease emergence, *E. fuscus* populations could be increasing following *Pd* introduction, particularly with more resources available to them following the loss of species highly susceptible to severe infections. The degree to which *E. fuscus* could be released from competition following the introduction of *Pd*, which drives species like *M. lucifugus*, *M. septentrionalis* and *P. subflavus* (tri‐colored bat) near extinction (Cheng et al., 2021), is unclear. However, increased capture rates of *E. fuscus* paired with decreased capture rates of species highly susceptible to severe *Pd* infection in the eastern US (Francl et al., 2012; O'Keefe et al., 2019; Pettit & O'Keefe, 2017; Thalken et al., 2018), could indicate indirect ecological consequences of white‐nose syndrome.

We found increased capture rates of *E. fuscus* across the eastern US after *Pd* invaded the landscape, possibly representing increases in relative abundance. Competitive release can occur when a species that overlaps niche space with another species is removed; the species that remains exploits more of that niche space and in turn, increases their abundance within that niche space (Wilson, 1961). Host species *M. lucifugus*, *M. septentrionalis* and *P. subflavus*, whose mortality rates and population declines from *Pd* are above 90% (Cheng et al., 2021), overlap their range, foraging and roosting habitat preferences (Agosta, 2002; Simonis et al., 2020), dietary preferences (Whitaker, 2004), and foraging and dietary niche space with *E. fuscus* in the eastern US (Jachowski et al., 2014; Wray & Peery, 2022). For example, *E. fuscus* and *M. lucifugus* in the midwestern US overlap dietary niche space, and that overlap has increased from 24% to 45% since 1898 (Wray & Peery, 2022). This increase of overlapping niche space over time is led by shifts in *E. fuscus'* dietary niche space becoming more similar to *M. lucifugus'* (Wray & Peery, 2022), and an ecological release event across *Pd* invasion time could contribute to that shift. Therefore, *E. fuscus* could be undergoing a disease‐mediated ecological release from competition following high mortality rates from species that overlap that niche space.

### 4.3 Abiotic mechanisms for increases to capture rates

Other factors that are not related to spatiotemporal disease spread may also contribute to increases in *E. fuscus* capture rates throughout the 30‐year period of this study, and could have additive effects with pathogen exposure time. For example, in the eastern US, capture rates of temperate bat species can be dependent on available foraging habitat such that, capture success in New Hampshire increased with overhanging canopy coverage above mist nets through forested corridors (Kunz & Brock, 1975). Temperate bat activity (including *E. fuscus*) also decreased with increasing precipitation and increased with increasing temperature in September – October in Ontario, Canada (Haddaway & McGuire, 2022). Within overlapping periods of time as the data presented here, increased precipitation was related to decreases in bat captures (including *E. fuscus*) in New Mexico, US (Geluso & Geluso, 2012). Taken together, foraging habitat availability, temperature and precipitation may all contribute to variation in *E. fuscus* capture rates in addition to ongoing disease pressures. Future research should incorporate climatic and landuse variables into capture rate models to determine if changing habitats and environmental factors have additive effects on *E. fuscus* populations and their compositions with long‐term pathogen exposure.

Annual precipitation also contributes to insect abundance, which would impact the number of bats captured while they are out foraging during mist net surveys. In the northeastern US, harsher winter conditions associated with climate change causes less snow accumulation and deeper soil freezing (Groffman et al., 2001), possibly contributing to declines in beetle abundance in the following spring and summers (Harris et al., 2019). Insect abundance is in global decline due to multiple factors associated with climate change such as drought, agricultural intensification and deforestation (Wagner et al., 2021). Therefore, if factors associated with climate change are further limiting insect abundance over time (which are *E. fuscus*' food resource), they may also contribute to behavioral changes in bat foraging frequency, our ability to capture bats with mist nets while they are foraging and thus, changes in *E. fuscus* capture rates over time and space. To determine how the interaction between precipitation and insect abundance changes *E. fuscus* capture rates, future research could test for changes to capture rates as functions of spatial insect abundance data and annual precipitation rates.

### 4.4 Reproductive mechanisms for compositional changes in capture rates by sex

Increases to female *E. fuscus* capture rates outpaced males over *Pd* invasion time. Greater increases to female capture rates compared to males could indicate a facultative adjustment of sex ratios via offspring (Cameron, 2004). This has been demonstrated in other species following the emergence of a pathogen. For example, female *Sarcophilus harrisii* (Tasmanian devils) infected with devil facial tumor disease have two times as many daughters as noninfected females, possibly due to changes in maternal condition (Lachish et al., 2009). Alternatively, it is also possible that male bats, particularly those less than a year old, may not survive hibernation when also confronted with *Pd* due to their annual reproductive cycle. Male *E. fuscus* become sexually mature by their first hibernation following birth and mate with females throughout late fall and winter (Christian, 1956; Kurta & Baker, 1990; Mumford, 1958; Phillips, 1966). Increased testosterone needed to reproduce during this time may suppress innate immunity (as seen in small rodents; Hughes & Randolph, 2001), which would be critical as a first line of defense against *Pd* infections for male *E. fuscus* in their first winter. Therefore, although the capture rates of male *E. fuscus* increased in spring through fall months from preinvasion to established years, decreased chances for survival in winter months could keep their capture rates from increasing as quickly as females. Further research following offspring sex ratios in *E. fuscus* maternity colonies, or identifying innate immune responses of *Pd* infected first year male bats, is needed to determine the mechanisms which underpin how the sex ratios of *E. fuscus* change with long‐term pathogen exposure.

The terminal investment hypothesis states that animals that reproduce multiple times in their lifespan should increase reproductive investment in the event that they are less likely to survive (Williams, 1966). In many disease systems, the terminal investment hypothesis is supported via increases in the number of offspring produced and/or decreases in age of first reproduction (Bonneaud et al., 2004; Giehr et al., 2017; Lachish et al., 2009). The overall capture rates of pregnant female *E. fuscus* increased in the eastern US from preinvasion to establishment years. These increases could also reflect decreases to the average age of first reproduction. While some female *E. fuscus* are able to reproduce in their first adult year, this is not the standard (Schowalter & Gunson, 1979). However, annual *Pd* exposure over many years could increase the number of pregnant 1‐year‐old female *E. fuscus*, regardless of reproductive success. In the white‐nose syndrome system, 1‐year‐old *M. lucifugus* increased yearling reproductive rates by nearly 74% following *Pd* introduction and their steep population declines (Ineson, 2020). Here, capture rates of pregnant *E. fuscus* almost doubled across the eastern US from preinvasion to establishment years. If *E. fuscus* follow a similar pattern to *M. lucifugus*, the overall increases to capture rates of pregnant female *E. fuscus* found here could provide supporting evidence for the terminal investment hypothesis. Further research examining known colonies of *E. fuscus* need to be performed to determine if 1‐year‐old reproductive rates have increased as a response to pathogen invasion.

The timing of reproductive investment can also change when an animal's environment is disrupted (Francl et al., 2012; Ineson, 2020; Roznik et al., 2015; Todd et al., 2011; Zurowski et al., 2020). For North American temperate bats, earlier pregnancy and pup births are thought to provide young of the year with more time to prepare for hibernation and thus, increase survival (Frick et al., 2010). Highly susceptible *M. lucifugus* become pregnant earlier following *Pd* introduction and give birth to pups earlier (Francl et al., 2012; Ineson, 2020), and similar shifts in reproductive timing could contribute to results for increases in the overall capture rates of reproductive *E. fuscus*. We found that pregnant and post‐lactating capture rates increased by *Pd* establishment, and lactating bat capture rates remained the same across disease time‐steps. Increases to post‐lactating bat capture rates could indicate that reproductive timing has shifted similar to *M. lucifugus* following *Pd* introduction. Further investigation is needed to determine whether changes in *E. fuscus* reproductive timing occur across a broad spatial scale with long‐term *Pd* exposure.

Other reproductive investment factors may contribute to the complexities of the capture rates these data demonstrate. Pathogen infections can also cause decreased parental care to offspring due to host energy trade‐offs between mounting immune responses to fight infections versus investing in reproduction (Bonneaud et al., 2003). For example, model estimates here indicate that pregnant bat capture rates increase by *Pd* establishment, but lactating bat capture rates do not change, suggesting that any increased investment toward reproduction may not be completely successful due to the lack of increases in capture rates at the pup‐rearing stage. Furthermore, post‐lactating capture rates more than doubled from preinvasion to establishment years, supporting similar patterns in Appalachian Regions of the US (O'Keefe et al., 2019). Increases to the capture rates of post‐lactating *E. fusucs* is concerning because they could potentially be transitioning into post‐lactation earlier in the maternal season, and diluting any increases to lactating capture rates that would be present in the field. This brings overall reproductive success into question and if (1) *E. fuscus* are abandoning pups, and/or (2) if newly flighted pups have enough maternal care in the summer to be successful in their first winter hibernation in combination with *Pd*. If female *E. fuscus* are transitioning into post‐lactation earlier, and pups are surviving but are not getting the full extent of needed maternal care, mortalities of young adult bats in their first hibernation could reflect why we see declines in *E. fuscus* winter populations (Cheng et al., 2021), but also see overall increases to capture rates in spring through fall months as presented here. To determine if *E. fuscus* are providing sufficient parental care with long‐term pathogen exposure, research quantifying reproductive success with juvenile *E. fuscus* survivorship, capture rates, body condition and growth is needed across a broader spatial scale over invasion time‐steps. Finally, year‐round estimates of *E. fuscus* host populations are needed to truly quantify population persistence and fecundity.

## 5 CONCLUSION

In conclusion, our work highlights the need to better understand how persisting host populations change across broad spatial scales with continued pathogen exposure. Persisting host population compositions can shift demographically and spatially with chronic pathogen exposure. Consequently, those spatial and demographic shifts over time could negatively impact the persistence of hosts like *E. fuscus* in the future if we do not aim to determine the ecological mechanism(s) behind broad patterns (i.e. cumulative energy deficits, competition, abiotic pressures, and/or reproductive investment pressures). Future efforts for understanding the mechanism(s) and degree of persistence of wildlife hosts like *E. fuscus* will be critical for understanding future population compositions of persisting host species following emerging infectious disease outbreaks and epidemics.

## AUTHOR CONTRIBUTIONS

**Molly C. Simonis:** Data curation (lead); formal analysis (lead); investigation (lead); methodology (lead); project administration (lead); visualization (lead); writing – original draft (lead); writing – review and editing (lead). **Lynn K. Hartzler:** Conceptualization (supporting); investigation (supporting); methodology (supporting); project administration (supporting); supervision (supporting); visualization (supporting); writing – original draft (supporting); writing – review and editing (supporting). **Gregory G. Turner:** Data curation (supporting); writing – review and editing (supporting). **Michael R. Scafini:** Data curation (supporting); writing – review and editing (supporting). **Joseph S. Johnson:** Writing – review and editing (supporting). **Megan A. Rúa:** Conceptualization (supporting); formal analysis (supporting); investigation (supporting); methodology (supporting); project administration (supporting); supervision (lead); visualization (supporting); writing – original draft (supporting); writing – review and editing (supporting).

## FUNDING INFORMATION

Data collection the Virginia for this publication was completed with funds provided by the Virginia Department of Wildlife Resources using resources from the National Wildlife Restoration Program provided by US Fish and Wildlife Service. Financial support for publication was provided by the University of Oklahoma Libraries’ Open Access Fund.

## CONFLICT OF INTEREST STATEMENT

There are no conflicts of interest to report for any author.

## Supporting information


Data S1:

## Data Availability

Data (Simonis et al., 2022; Simonis, Hartzler, Campbell, et al., 2023) are available from Dryad Digital Repository at https://doi.org/10.5061/dryad.ngf1vhhvv. R code used for all analyses in this manuscript are available on Simonis' GitHub page at https://github.com/simonimc. R code used for analyses are not novel and use functions and packages that are well established.
