# Supplementary material for: Capture rates of Eptesicus fuscus increase following white‐nose syndrome across the eastern US
Source: Ecol Evol. 2024 Jun 25;14(6):e11523. doi: 10.1002/ece3.11523 (PMC11199122; doi:10.1002/ece3.11523)
Supplement: Supplementary file 1 — Data S1: [file ECE3-14-e11523-s001.docx]

**Supporting Information**

**Journal Name:** *Ecology and Evolution*

**Manuscript Type:** Article

**Manuscript Title:**  Capture rates of persisting *Eptesicus fuscus* increase following white-nose syndrome across the eastern US

**Authors:** Molly C Simonis^1,2^, Lynn K Hartzler^1,3^, Greg G Turner^4^, Michael R Scafini^4^, Joseph S Johnson^5^, and Megan A Rúa^1,3^

1 Wright State University Environmental Sciences PhD Program, Dayton, OH, USA

2 University of Oklahoma Department of Biology, Norman, OK, USA

3 Wright State University Department of Biological Sciences, Dayton, OH, USA

4 Pennsylvania Game Commission Bureau of Wildlife Management, Harrisburg, PA, USA

5 University of Cincinnati School of Information Technology, Cincinnati, OH, USA

**Corresponding author:** Molly C Simonis, [molly.simonis@ou.edu](mailto:molly.simonis@ou.edu)

**Open Research Statement:** Data (Simonis et al., 2022) are available from Dryad Digital Repository at <https://doi.org/10.5061/dryad.ngf1vhhvv>. R code used for all analyses in this manuscript are available on Simonis’ GitHub page at <https://github.com/simonimc>. R code used for analyses are not novel, and use functions and packages that are well known.

Table S1. Estimated conditional effects of adult and female-only Bayesian Generalized Linear Models when latitude is a continuous variable.

| **Model** | **sex** | ***Pd* time-step** | **Estimated Latitudinal Slope** | **lower 95% credible interval** | **upper 95% credible interval** |
| --- | --- | --- | --- | --- | --- |
| Adult | female | pre-invasion | -0.01 | -0.03 | 0.01 |
|  | female | invasion | 0.00 | -0.02 | 0.02 |
|  | female | epidemic | -0.02 | -0.03 | 0.00 |
|  | female | established | 0.01 | -0.01 | 0.03 |
|  | male | pre-invasion | 0.00 | -0.03 | 0.02 |
|  | male | invasion | 0.02 | 0.00 | 0.03 |
|  | male | epidemic | -0.02 | -0.04 | -0.01 |
|  | male | established | 0.01 | -0.01 | 0.03 |
| **Model** | **reproductive status** | ***Pd* time-step** | **Estimated Latitudinal Slope** | **lower 95% credible interval** | **upper 95% credible interval** |
| Female-only | non-reproductive | pre-invasion | -0.05 | -0.07 | -0.02 |
|  | non-reproductive | invasion | -0.05 | -0.07 | -0.02 |
|  | non-reproductive | epidemic | -0.06 | -0.08 | -0.03 |
|  | non-reproductive | established | -0.04 | -0.07 | -0.01 |
|  | pregnant | pre-invasion | -0.03 | -0.07 | 0.00 |
|  | pregnant | invasion | -0.02 | -0.06 | 0.02 |
|  | pregnant | epidemic | -0.04 | -0.07 | -0.01 |
|  | pregnant | established | -0.02 | -0.06 | 0.02 |
|  | lactating | pre-invasion | 0.00 | -0.04 | 0.03 |
|  | lactating | invasion | -0.01 | -0.04 | 0.02 |
|  | lactating | epidemic | -0.02 | -0.04 | 0.01 |
|  | lactating | established | 0.02 | -0.02 | 0.05 |
|  | post-lactating | pre-invasion | 0.00 | -0.03 | 0.03 |
|  | post-lactating | invasion | 0.03 | 0.00 | 0.05 |
|  | post-lactating | epidemic | 0.02 | 0.00 | 0.05 |
|  | post-lactating | established | 0.03 | 0.00 | 0.06 |

Table S2. Estimated conditional effects of adult and female-only Bayesian Generalized Linear Models when latitude is a categorical variable for ‘north’ or ‘south’ of the spatial threshold at 39.3 °N.

| **Model** | **sex** | ***Pd* time-step** | **latitudinal category** | **estimate** | **lower 95% credible interval** | **upper 95% credible interval** |
| --- | --- | --- | --- | --- | --- | --- |
| Adults | female | ***---*** | ***---*** | 2.62 | 2.47 | 2.78 |
|  | male | ***---*** | ***---*** | 1.85 | 1.75 | 1.97 |
|  | ***---*** | pre-invasion | ***---*** | 2.02 | 1.81 | 2.26 |
|  | ***---*** | invasion | ***---*** | 2.02 | 1.83 | 2.24 |
|  | ***---*** | epidemic | ***---*** | 1.96 | 1.82 | 2.11 |
|  | ***---*** | established | ***---*** | 2.94 | 2.70 | 3.19 |
|  | ***---*** | ***---*** | north | 2.17 | 2.03 | 2.32 |
|  | ***---*** | ***---*** | south | 2.24 | 2.07 | 2.41 |
|  | female | pre-invasion | ***---*** | 2.20 | 1.93 | 2.51 |
|  | male | pre-invasion | ***---*** | 1.86 | 1.62 | 2.13 |
|  | female | invasion | ***---*** | 2.60 | 2.29 | 2.94 |
|  | male | invasion | ***---*** | 1.57 | 1.39 | 1.78 |
|  | female | epidemic | ***---*** | 2.34 | 2.14 | 2.56 |
|  | male | epidemic | ***---*** | 1.64 | 1.50 | 1.80 |
|  | female | established | ***---*** | 3.50 | 3.15 | 3.89 |
|  | male | established | ***---*** | 2.47 | 2.22 | 2.74 |
|  | female | ***---*** | north | 2.54 | 2.36 | 2.75 |
|  | female | ***---*** | south | 2.69 | 2.45 | 2.95 |
|  | male | ***---*** | north | 1.85 | 1.71 | 2.00 |
|  | male | ***---*** | south | 1.86 | 1.70 | 2.04 |
|  | ***---*** | pre-invasion | north | 1.86 | 1.55 | 2.23 |
|  | ***---*** | pre-invasion | south | 2.20 | 1.96 | 2.49 |
|  | ***---*** | invasion | north | 2.08 | 1.85 | 2.32 |
|  | ***---*** | invasion | south | 1.97 | 1.67 | 2.33 |
|  | ***---*** | epidemic | north | 1.80 | 1.65 | 1.96 |
|  | ***---*** | epidemic | south | 2.14 | 1.89 | 2.40 |
|  | ***---*** | established | north | 3.20 | 2.91 | 3.51 |
|  | ***---*** | established | south | 2.70 | 2.35 | 3.10 |
|  | female | pre-invasion | north | 1.94 | 1.57 | 2.40 |
|  | female | invasion | north | 2.55 | 2.22 | 2.92 |
|  | female | epidemic | north | 2.21 | 2.00 | 2.46 |
|  | female | established | north | 3.82 | 3.41 | 4.28 |
|  | female | pre-invasion | south | 2.50 | 2.15 | 2.92 |
|  | female | invasion | south | 2.64 | 2.14 | 3.24 |
|  | female | epidemic | south | 2.48 | 2.14 | 2.86 |
|  | female | established | south | 3.19 | 2.69 | 3.82 |
|  | male | pre-invasion | north | 1.78 | 1.42 | 2.25 |
|  | male | invasion | north | 1.69 | 1.47 | 1.95 |
|  | male | epidemic | north | 1.46 | 1.30 | 1.61 |
|  | male | established | north | 2.67 | 2.38 | 3.00 |
|  | male | pre-invasion | south | 1.94 | 1.67 | 2.27 |
|  | male | invasion | south | 1.46 | 1.19 | 1.79 |
|  | male | epidemic | south | 1.84 | 1.59 | 2.14 |
|  | male | established | south | 2.28 | 1.92 | 2.72 |
| **Model** | **reproductive status** | ***Pd* time-step** | **latitudinal category** | **estimate** | **lower 95% credible interval** | **upper 95% credible interval** |
| Female-only | non-reproductive | **---** | **---** | 2.21 | 1.99 | 2.46 |
|  | pregnant | **---** | **---** | 3.88 | 3.43 | 4.42 |
|  | lactating | **---** | **---** | 5.43 | 4.95 | 5.99 |
|  | post-lactating | **---** | **---** | 4.73 | 4.30 | 5.18 |
|  | **---** | pre-invasion | **---** | 3.28 | 2.86 | 3.76 |
|  | **---** | invasion | **---** | 4.00 | 3.49 | 4.56 |
|  | **---** | epidemic | **---** | 3.42 | 3.10 | 3.76 |
|  | **---** | established | **---** | 4.91 | 4.38 | 5.47 |
|  | **---** | **---** | north | 3.72 | 3.43 | 4.05 |
|  | **---** | **---** | south | 3.99 | 3.63 | 4.38 |
|  | non-reproductive | pre-invasion | **---** | 2.24 | 1.80 | 2.84 |
|  | pregnant | pre-invasion | **---** | 2.91 | 2.17 | 3.90 |
|  | lactating | pre-invasion | **---** | 5.02 | 3.98 | 6.24 |
|  | post-lactating | pre-invasion | **---** | 3.54 | 2.88 | 4.34 |
|  | non-reproductive | invasion | **---** | 2.35 | 1.87 | 2.99 |
|  | pregnant | invasion | **---** | 3.68 | 2.83 | 4.87 |
|  | lactating | invasion | **---** | 5.73 | 4.70 | 6.97 |
|  | post-lactating | invasion | **---** | 5.18 | 4.31 | 6.27 |
|  | non-reproductive | epidemic | **---** | 1.81 | 1.53 | 2.17 |
|  | pregnant | epidemic | **---** | 4.17 | 3.45 | 4.99 |
|  | lactating | epidemic | **---** | 4.53 | 3.92 | 5.23 |
|  | post-lactating | epidemic | **---** | 4.00 | 3.45 | 4.64 |
|  | non-reproductive | established | **---** | 2.50 | 2.07 | 3.02 |
|  | pregnant | established | **---** | 5.11 | 3.99 | 6.44 |
|  | lactating | established | **---** | 6.64 | 5.63 | 7.77 |
|  | post-lactating | established | **---** | 6.83 | 5.72 | 8.12 |
|  | non-reproductive | **---** | north | 1.76 | 1.53 | 2.02 |
|  | pregnant | **---** | north | 3.37 | 2.83 | 4.02 |
|  | lactating | **---** | north | 5.98 | 5.32 | 6.74 |
|  | post-lactating | **---** | north | 5.39 | 4.80 | 6.03 |
|  | non-reproductive | **---** | south | 2.77 | 2.36 | 3.26 |
|  | pregnant | **---** | south | 4.48 | 3.72 | 5.36 |
|  | lactating | **---** | south | 4.92 | 4.26 | 5.71 |
|  | post-lactating | **---** | south | 4.15 | 3.57 | 4.79 |
|  | **---** | pre-invasion | north | 3.17 | 2.56 | 3.96 |
|  | **---** | invasion | north | 3.74 | 3.22 | 4.36 |
|  | **---** | epidemic | north | 3.15 | 2.81 | 3.53 |
|  | **---** | established | north | 5.12 | 4.54 | 5.75 |
|  | **---** | pre-invasion | south | 3.39 | 2.88 | 3.98 |
|  | **---** | invasion | south | 4.29 | 3.46 | 5.32 |
|  | **---** | epidemic | south | 3.71 | 3.19 | 4.33 |
|  | **---** | established | south | 4.70 | 3.90 | 5.66 |
|  | non-reproductive | pre-invasion | north | 2.03 | 1.41 | 2.89 |
|  | non-reproductive | invasion | north | 1.77 | 1.33 | 2.34 |
|  | non-reproductive | epidemic | north | 1.33 | 1.09 | 1.63 |
|  | non-reproductive | established | north | 2.02 | 1.62 | 2.50 |
|  | non-reproductive | pre-invasion | south | 2.47 | 1.91 | 3.28 |
|  | non-reproductive | invasion | south | 3.13 | 2.17 | 4.57 |
|  | non-reproductive | epidemic | south | 2.47 | 1.87 | 3.27 |
|  | non-reproductive | established | south | 3.10 | 2.28 | 4.23 |
|  | pregnant | pre-invasion | north | 2.42 | 1.51 | 4.02 |
|  | pregnant | invasion | north | 3.33 | 2.45 | 4.62 |
|  | pregnant | epidemic | north | 3.32 | 2.57 | 4.31 |
|  | pregnant | established | north | 4.81 | 3.83 | 6.08 |
|  | pregnant | pre-invasion | south | 3.49 | 2.54 | 4.78 |
|  | pregnant | invasion | south | 4.06 | 2.65 | 6.34 |
|  | pregnant | epidemic | south | 5.23 | 4.06 | 6.81 |
|  | pregnant | established | south | 5.43 | 3.59 | 8.13 |
|  | lactating | pre-invasion | north | 5.99 | 4.25 | 8.46 |
|  | lactating | invasion | north | 5.62 | 4.57 | 6.96 |
|  | lactating | epidemic | north | 4.58 | 3.91 | 5.33 |
|  | lactating | established | north | 8.30 | 7.02 | 9.75 |
|  | lactating | pre-invasion | south | 4.21 | 3.13 | 5.51 |
|  | lactating | invasion | south | 5.85 | 4.15 | 8.10 |
|  | lactating | epidemic | south | 4.48 | 3.52 | 5.70 |
|  | lactating | established | south | 5.32 | 4.03 | 6.98 |
|  | post-lactating | pre-invasion | north | 3.44 | 2.46 | 4.71 |
|  | post-lactating | invasion | north | 5.91 | 4.86 | 7.21 |
|  | post-lactating | epidemic | north | 4.87 | 4.24 | 5.63 |
|  | post-lactating | established | north | 8.55 | 7.13 | 10.30 |
|  | post-lactating | pre-invasion | south | 3.64 | 2.84 | 4.68 |
|  | post-lactating | invasion | south | 4.54 | 3.28 | 6.23 |
|  | post-lactating | epidemic | south | 3.28 | 2.54 | 4.26 |
|  | post-lactating | established | south | 5.46 | 4.03 | 7.33 |


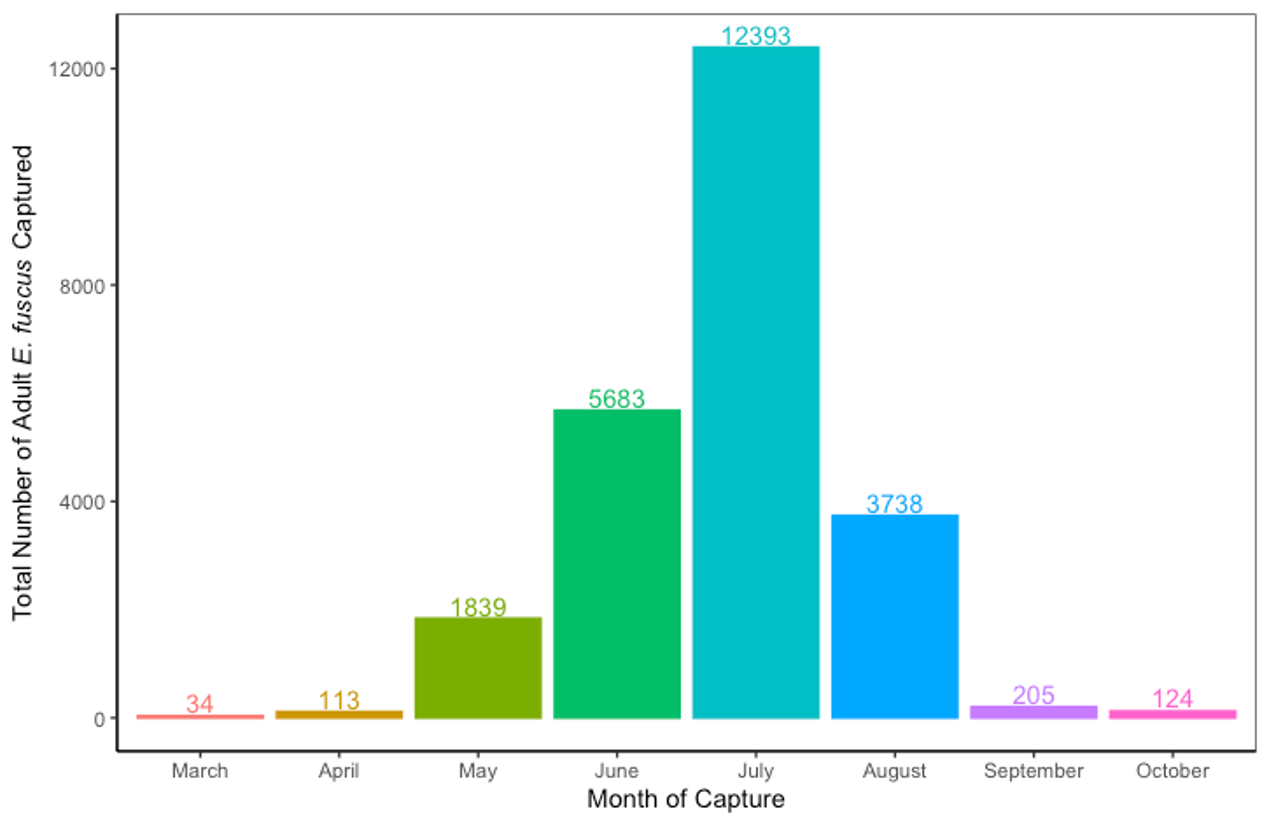


Figure S1. Total number of adult *E. fuscus* captured each month within the 30-year capture dataset used for analyses in this manuscript (Simonis et al. 2022, 2023b, 2023a).

**
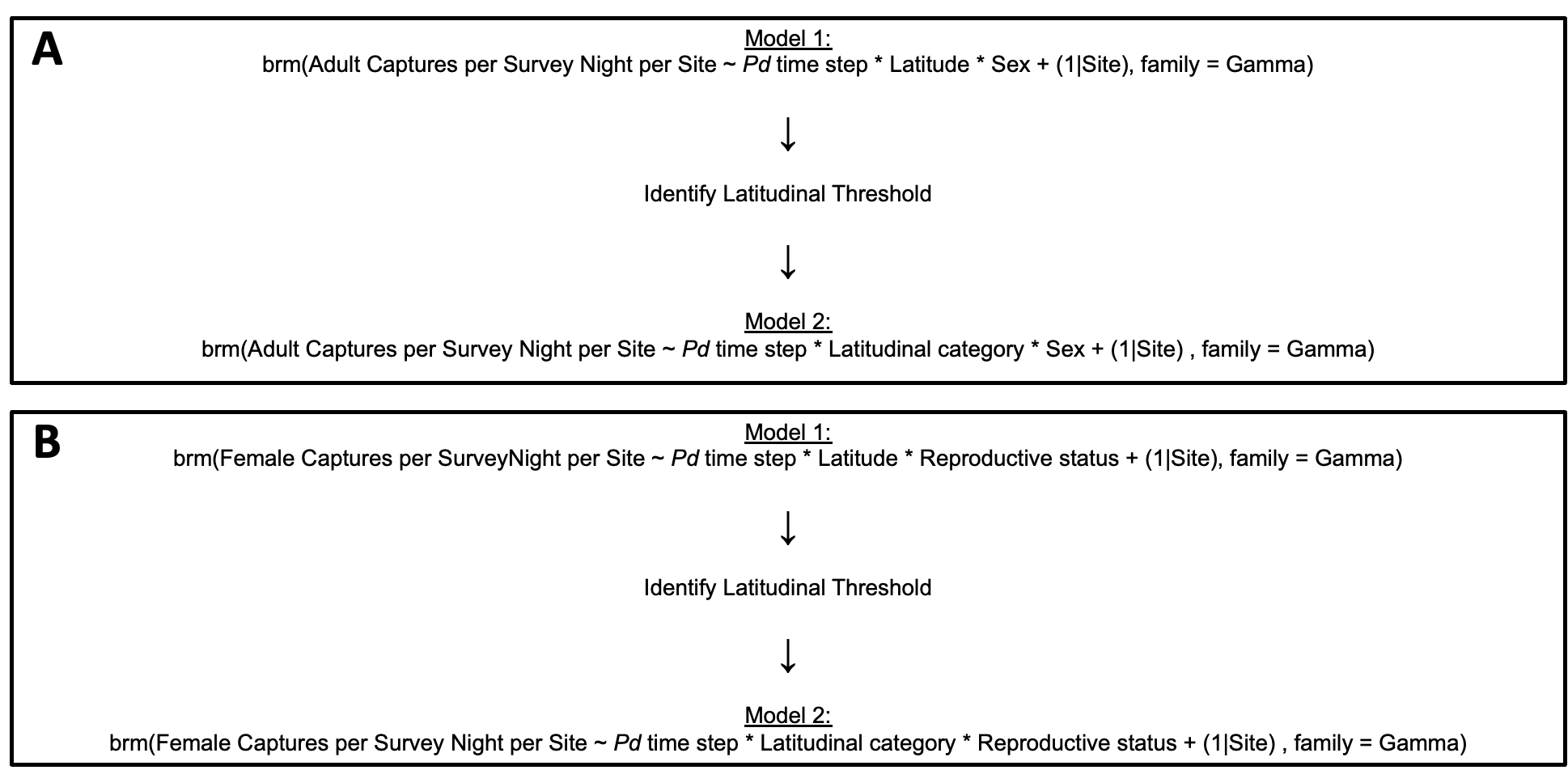
**

Figure S2. Bayesian generalized linear model creation occurred in two steps to determine differences in adult demographics (A) or female reproductive demographics (non-reproductive, pregnant, lactating and post-lactating) (B). Models were created using the *brms* package in R (Bürkner 2017; R Core Team 2021). *Pseudogymnoascus destructans* (*Pd*) time-steps were categorical for the number of years since suspected or confirmed *Pd* introduction within each state of capture and included pre-invasion (< 0 years with *Pd*), invasion (0-1 years with *Pd*), epidemic (2-4 years with *Pd*) and established years (5+ years with *Pd*).


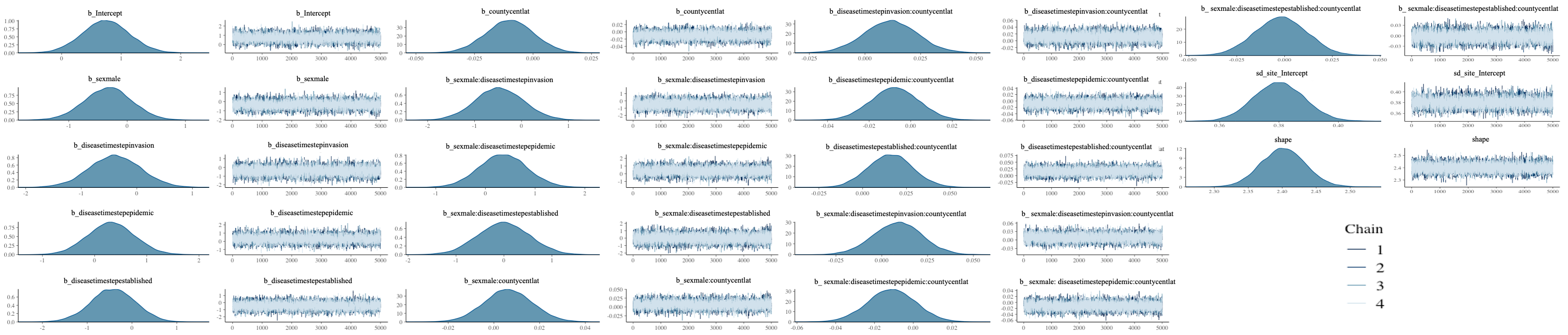


Figure S3. Convergence plots for the initial adult *Eptesicus fuscus* Bayesian Generalized Linear Model where the number of *E. fuscus* captures per survey night per site were a function of a conditional effects interaction between continuous values for county centroid latitude of capture, sex (male/female) and *Pseudogymnoascus destructans* disease time-steps (pre-invasion/invasion/epidemic/established) and a marginal effect of site.


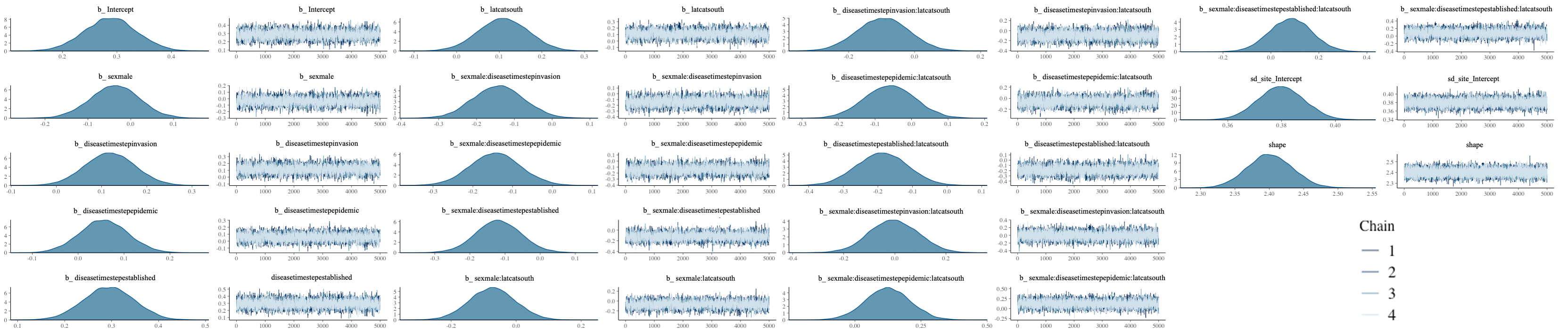


Figure S4. Convergence plots for the second adult *Eptesicus fuscus* Bayesian Generalized Linear Model where the number of *E. fuscus* captures survey per night per site were a function of a latitudinal category (north/south of 39.5 °N), sex (male/female) and *Pseudogymnoascus destructans* disease time-steps (pre-invasion/invasion/epidemic/established) and a marginal effect of site.


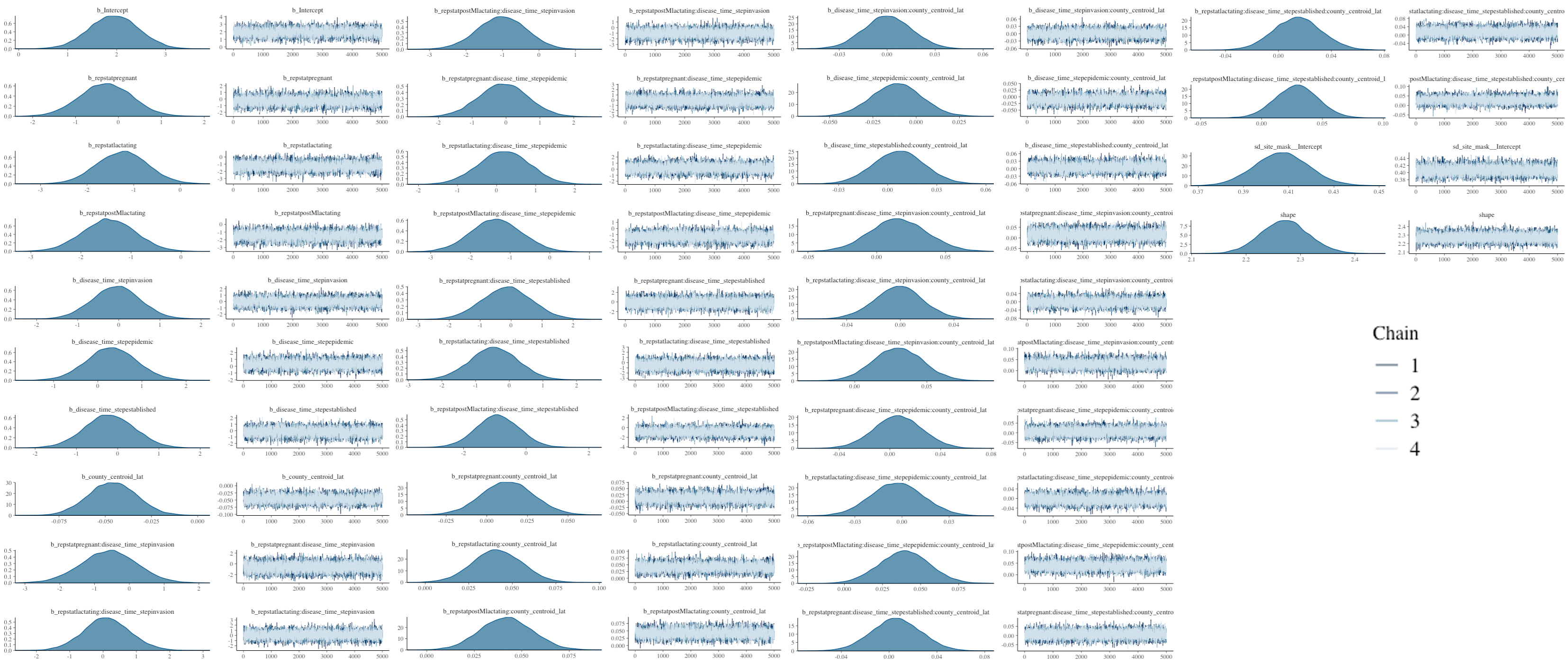


Figure S5. Convergence plots for the initial female-only *Eptesicus fuscus* Bayesian Generalized Linear Model where the number of adult female *E. fuscus* captures per survey night per site were a function of continuous values for county centroid latitude of capture, reproductive status (non-reproductive/pregnant/lactating/post-lactating) and *Pseudogymnoascus destructans* disease time-steps (pre-invasion/invasion/epidemic/established), and a marginal effect of site.


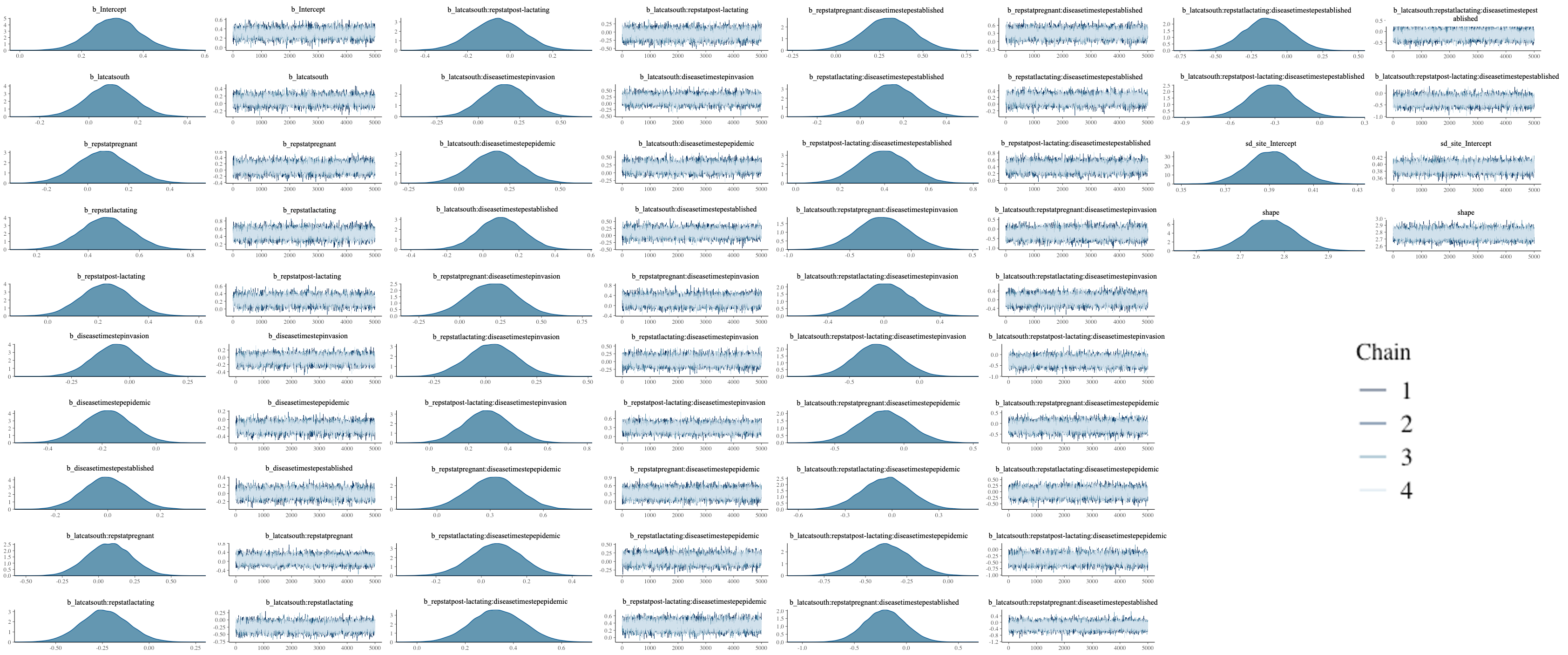


Figure S6. Convergence plots for the second female-only *Eptesicus fuscus* Bayesian Generalized Linear Model where the number of adult female *E. fuscus* captures per survey night per site were a function of a latitudinal category (north/south of 39.5 °N), reproductive status (non-reproductive/pregnant/lactating/post-lactating) and *Pseudogymnoascus destructans* disease time-steps (pre-invasion/invasion/epidemic/established) and a marginal effect of site.

**References**

Simonis, M. C., L. K. Hartzler, J. Campbell, T. C. Carter, L. N. Cooper, K. Cross, K. Etchison, T. Hemberger, R. A. King, R. Reynolds, Y. Samar, M. R. Scafini, S. Stankavich, G. G. Turner, and M. A. Rúa. 2023a. Long-term spring through fall capture data of *Eptesicus fuscus* in the eastern USA before and after white-nose syndrome. Data in Brief 49:109353. <https://doi.org/10.1016/j.dib.2023.109353>

Simonis, M. C., L. K. Hartzler, J. S. Johnson, G. G. Turner, M. R. Scafini, and M. A. Rúa. 2022. Big brown bat (*Eptesicus fuscus*) capture records before and after white-nose syndrome. Dryad, Dataset. <https://doi.org/10.5061/dryad.ngf1vhhvv>

Simonis, M. C., L. K. Hartzler, G. G. Turner, M. R. Scafini, J. S. Johnson, and M. A. Rúa. 2023b. Long-term exposure to an invasive fungal pathogen decreases *Eptesicus fuscus* body mass with increasing latitude. Ecosphere 14:e4426. <https://doi.org/10.1002/ecs2.4426>
